# Depth-Keeping Control for a Deep-Sea Self-Holding Intelligent Buoy System Based on Inversion Time Constraint Stability Strategy Optimization

**DOI:** 10.3390/s22031096

**Published:** 2022-01-31

**Authors:** Qiang Wang, Xingfei Li, Zurong Qiu, Shizhong Yang, Wei Zhou, Jingbo Zhao

**Affiliations:** 1 School of Information and Control Engineering, Qingdao University of Technology, Qingdao 266525, China; gavinwang_qut@163.com (Q.W.); yangszqut@163.com (S.Y.); zhouwqut@163.com (W.Z.); 2 State Key Laboratory of Precision Measuring Technology and Instruments, Tianjin University, Tianjin 300072, China; lxingfei_tju@163.com (X.L.); qzrtju@163.com (Z.Q.); 3 Qingdao Institute for Ocean Technology of Tianjin University, Qingdao 266200, China

**Keywords:** deep-sea self-holding intelligent buoy (DSIB), inversion time-constraint stability (ITCS), quantum genetic algorithm (QGA), chaotic particle swarm optimization (CPSO), depth control

## Abstract

Based on the nonlinear disturbance observer (NDO), the inversion time-constraint stability strategy (ITCS) is designed to make the deep-sea self-holding intelligent buoy (DSIB) system hovered at an appointed depth within a specified time limit. However, it is very challenging to determine the optimal parameters of an ITCS depth controller. Firstly, a genetic algorithm based on quantum theory (QGA) is proposed to obtain the optimal parameter combination by using the individual expression form of quantum bit and the adjustment strategy of quantum rotary gate. To improve the speed and accuracy of global search in the QGA optimization process, taking the number of odd and even evolutions as the best combination point of the genetic and chaos particle swarm algorithm (GACPSO), an ITCS depth controller based on GACPSO strategy is proposed. Besides, the simulations and hardware-in-the-loop system experiments are conducted to examine the effectiveness and feasibility of the proposed QGA–ITCS and GACPSO–ITCS depth controller. The results show that the proposed GACPSO–ITCS depth controller provides higher stability with smaller steady-state error and less settling time in the depth-control process. The research of the proposed method can provide a stable operation condition for the marine sensors carried by the DSIB.

## 1. Introduction

**Figure 1:**
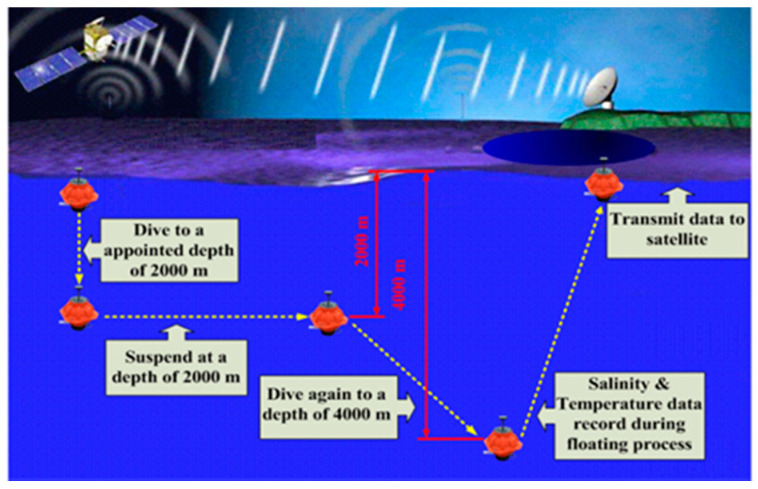
Schematic diagram of DSIB operation cycle profile measurement.

**Figure 2:**
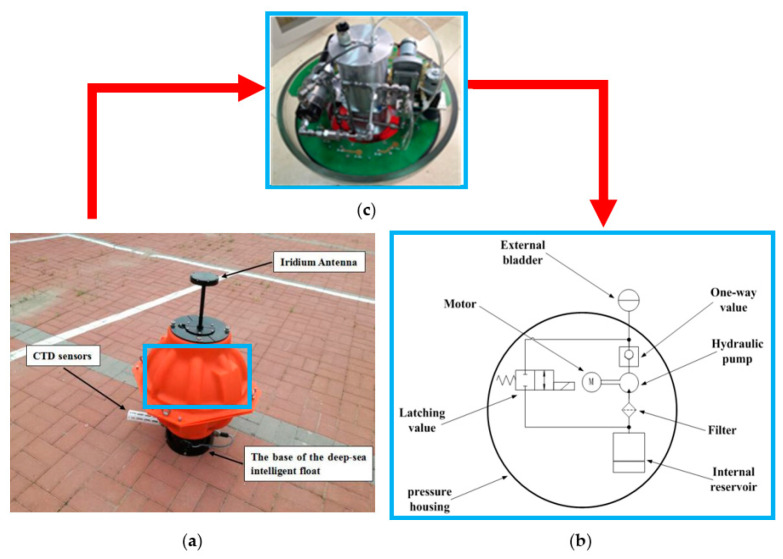
(**a**) The appearance drawing of a DSIB system prototype (The blue box represents the depth-positioning buoyancy adjustment system position of the DSIB); the (**b**) basic schematic diagram of a depth-positioning buoyancy adjustment system of the DSIB system; and (**c**) the actual internal structure photo of the depth-positioning buoyancy adjustment system.

With the intensification of global warming and the greenhouse effect, as well as the deterioration of the marine climate and environment, extreme weather events have been occurring with startling frequency. There are many uncertainties and challenges for the development and utilization of the ocean resources. Thus, marine environment monitoring can be employed not only for marine-climate models, marine disaster prediction, and weather forecasting, but also for fishery production, which provides help for analyzing and forecasting the formation, migration, and movement of marine fisheries, so as to guide people in their fishery production activities [1,2]. As a kind of ocean observation platform, a deep-sea self-holding intelligent buoy (DSIB) has been applied for ocean observation. The observation scheme of DSIB is shown in Figure 1 [3,4]. It enjoys the advantages of small size, convenient deployment, independent observation, and low price. The depth positioning of the DSIB is accomplished by volumetric change instead of weight change. The floating process is achieved by injecting hydraulic oil which is filled in an internal reservoir inside the spherical hull to the external bladder connected to the hydraulic pump and latching value. The exterior of the spherical hull is provided with an external bladder. The submerging process is realized by transporting hydraulic oil from the external oil bladder to the internal reservoir. The DSIB can dive or ascend to the target depth by changing the volume of external bladder. A schematic of the depth positioning buoyancy adjustment system and the appearance of the DSIB are shown in Figure 2. The depth positioning research of the DSIB can help us to construct ocean-related statistical models more accurately and effectively, and to better understand the ocean circulation and seawater energy exchange, and to know the laws deeper, contributing good service to human society. Thus, the depth-keeping control strategy at a specified depth is essential. Many researchers have extensively investigated the control methods for depth tracking and hovering of an underwater vehicle, and their achievements have been acknowledged in the current literature.

In terms of the principle of traditional PD method, an improved double PD depth-control strategy of the DSIB was proposed [5]. The DSIB suspended at a depth of 1000 m with a mean depth-tracking error within 6 m. Because the DSIB system model is established by means of the speed and flow expectation functions of the arctangent model, it was difficult to optimize the depth-control method. A state feedback depth controller coupled with an Extended Kalman Filter (EKF) was applied to the depth-positioning process of a profiling float [6]. However, the established dynamic model was only based on a more realistic compressibility law. There was no sufficiently detailed data in the literature to make an accurate comparison. The inversion controller and time-constraint stability controller have distinctive advantages of simple structure, easily-realized high precision, and strong robustness, which already presented some successful practical realizations [7,8,9,10]. To achieve time-constraint convergence of the tracking errors of Unmanned Underwater Vehicles (UUV), Josué et al. proposed a model-free high order sliding mode controller with a time-base generator. Simulation results showed that the designed controller can achieve the desired trajectory at the finite time. As the less accurate control design model is obtained whose shortcomings are compensated for using a suitable robust control method, the comprehensive system model was used to design a finite-time robust tracking control (FTRTC) of the autonomous underwater vehicle (AUV) model dynamically [8]. The overall system stability was demonstrated via Lyapunov theory through simulations. Cho et al. estimated the water flow and the external interference of AUV through time-delay estimation (TDE), and proposed to adopt the inversion method to design a controller to realize the depth-trajectory control process of the AUV [9]. By adopting the inversion-control strategy, the control input of the AUV can change smoothly and the peak value is small, which can avoid the overshoot, or “bottom touch”, problem when the AUV is diving. However, due to the high derivative of the virtual control quantity in the inversion method design, the calculation of the controller is more complex. In order to improve the noise attenuation performance, Huy et al. proposed an inversion controller based on the nonlinear disturbance observer (NDO) of the AUV [10]. The compensation input provided by NDO in the case of external disturbance was efficient. A depth error of 1 m was produced within 20 s in the depth-tracking process. However, the whole performance of the aforementioned inversion controller and time-constraint stability controller were not put into practice [7,8,9,10]. The settling time and depth error need to be improved by tuning the aforementioned controller parameters.

In the previous methods, the control effect of the depth controller mainly depends on the depth controller parameters setting, while the depth controller parameters setting is ultimately based on error feedback to eliminate, or minimize, system error. Depth controller parameters optimization is important and of great significance for stability, reliability, and fast response characteristics of the system. To meet the need of the increasingly complex depth-control process, a series of controller parameter setting strategies had become the key to improve and optimize the control performance. The optimization algorithm for controller parameters was viewed as a search of search population in the search space, the integration for absolute error, and the squared control input were used as optimization goals, and the optimal control quantity was calculated through iterative optimization. The controller parameters were taken as a search team. The optimization goals of parameter settings were realized by the particle swarm optimization (PSO) or genetic algorithm (GA). Tumari et al. proposed the PSO approach in optimizing the performance of the model-free PID depth controller with derivative filter applying in stabilizing a Hovering Autonomous Underwater Vehicle (HAUV) [11]. The simulation results showed that when the HAUV hovers at 3 m, the convergence time of the optimized depth controller is reduced by 66.7% compared with the pre-optimized depth controller. But the performance PSO tuning can be further improved by increasing the number of particles and iterations. To solve the premature convergence and getting stuck in the local minima of the PSO approach, a novel hybrid PSO algorithm, combining Sine Cosine Algorithm (SCA) and Levy Flight (LF) distribution, was used to optimize the gains of the PID depth controller [12]. The obtained simulation results demonstrate that the proposed PSOSCALF-tuned PID has higher accuracy and a faster convergence rate as compared to the PSO-based PID. In addition, the cloud-model-based quantum genetic algorithm (CQGA) was employed to tune coefficients of the fractional-order PID controller to improve performance of the AUV motion [13]. Through simulation analysis, it can be seen that when hovering control at a depth of 2.5 m, the depth error of the optimized fractional-order PID controller based on CQGA is reduced by 51.2% and the settling time is shortened by 25% compared with the fractional-order PID depth controller before optimization. However, the CQGA algorithm needs to complete complex coding and decoding in the operation process, which requires high operation speed of the system. This method should be tested on various depth-control scenarios of an actual system in future works.

Comparing the advantages and disadvantages of various depth-control strategies [5,6,7,8,9,10], it can be seen that the inversion method has been widely used in underwater vehicles motion control technology and achieved good results. At the same time, the time-constraint stability strategy has strong robustness and better anti-disturbance performance, and the state of the system can converge to the equilibrium state in a finite time. Motivated by the aforementioned analysis, the present study focuses on designing the depth-keeping controller for the vertical motion of a DSIB. The control strategies are produced by combining the inversion technique and the time-constraint stability law with the NDO to compensate for the errors caused by system interference. The anti-disturbance performance of the DSIB dynamical system can be made better. The inversion time-constraint stability strategy (ITCS) is suitable for solving depth-control problems. As the parameters of the ITCS need to be obtained based on experience data, some unexpected depth-keeping errors occur in different depth positioning conditions. The required depth positioning accuracy cannot be fulfilled considering the adaptability of the ITCS. ITCS depth controller parameter tuning is essential for the complex problems of strong coupling, multivariables, and multi-constraints. In practical application, too many parameters, unknown tuning range, and direction make it difficult to set parameters and limit the usage of ITCS. However, the tuning methodology of the aforementioned depth controllers highly depends on the operation iteration ability of the system, resulting in long implementation time [11,12,13]. In order to solve the problem of repeated adjustments in the parameter setting process of the ITCS depth controller and the possibility of non-optimal parameters, the new tuning technique is employed for the adjustment of key control parameters of the ITCS strategy for DSIB depth-control system. Firstly, the QGA (quantum genetic algorithm) is applied to the ITCS depth-controller parameters optimization in a systematic way. As an optimization algorithm, the quantum crossover, quantum mutation, and population catastrophe are introduced to the QGA–ITCS, carrying out the evolution computation to transform parameter tuning into parameter optimization. In order to solve the problems of high space consumption and low speed and accuracy of global optimization in the parameter optimization process of the QGA algorithm, a novel GACPSO–ITCS hybrid algorithm is proposed, combining the strengths of chaotic particle swarm optimization (CPSO) with genetic algorithm (GA) in this paper. GACPSO–ITCS combines the updated rules of velocity and situation of CPSOs with the ideas of selection, crossover, and mutation from GA. The organization of the rest of this paper is as follows: In Section 2, the drag coefficients of the DSIB model are estimated by the CFD simulations. In Section 3, based on the obtained drag coefficients, the dynamic model of DSIB is established. In Section 4, combining with the NDO, the ITCS depth controller is designed to observe and compensate for the disturbance. The search process of the proposed QGA–ITCS and GACAPSO–ITCS algorithms are designed to obtain the optimal parameter combination of the ITCS depth controller. In Section 5, relevant simulations and hardware-in-the-loop system experimental results demonstrate the applicability of the proposed QGA–ITCS and GACPSO–ITCS optimization method in various scenarios. Finally, some conclusions are drawn in Section 6.

## 2. Estimation and Analysis of the DSIB’s Hydrodynamics

The hydrodynamic analysis of the DSIB, namely the estimation of the drag coefficients, is required for accurate motion modeling, which, in turn, can provide necessary conditions for the depth-control simulation.

### 2.1. Determination of Flow Regime

Before the analysis of the drag force is carried out on the DSIB, the flow regime of the seawater needs be determined by Reynolds number value Re when the DSIB is ascending or diving. Reynolds number is expressed as follows [14]:

(1) Re=Dvρ/μ

where *D* is the diameter of the spherical pressure hull, *D* = 0.432 m. *μ* is the dynamic coefficient of viscosity, $\mu =1.792\times 10^{-3}\;\mathrm{Pa}\cdot s$. *ρ* is the density of seawater. Because the density of seawater increases with the diving depth, the range of the seawater density changes is about 2%. The average seawater density $\bar{\rho }$ is chosen to calculate the Reynolds number in this paper. $\rho =\bar{\rho }=1.034\times 10^{3}\;\mathrm{kg}/m^{3}$. *v* is the motion velocity of the DSIB. The mean motion velocity of the DSIB is selected as 0.145 m/s when the DSIB ascending or diving. Through calculation, Re=36,143.86>13,800. Because the obtained Reynolds number is above the critical Reynolds number the turbulence model is adopted when the DSIB is ascending or diving.

### 2.2. Flow Field Analysis

**Figure 3:**
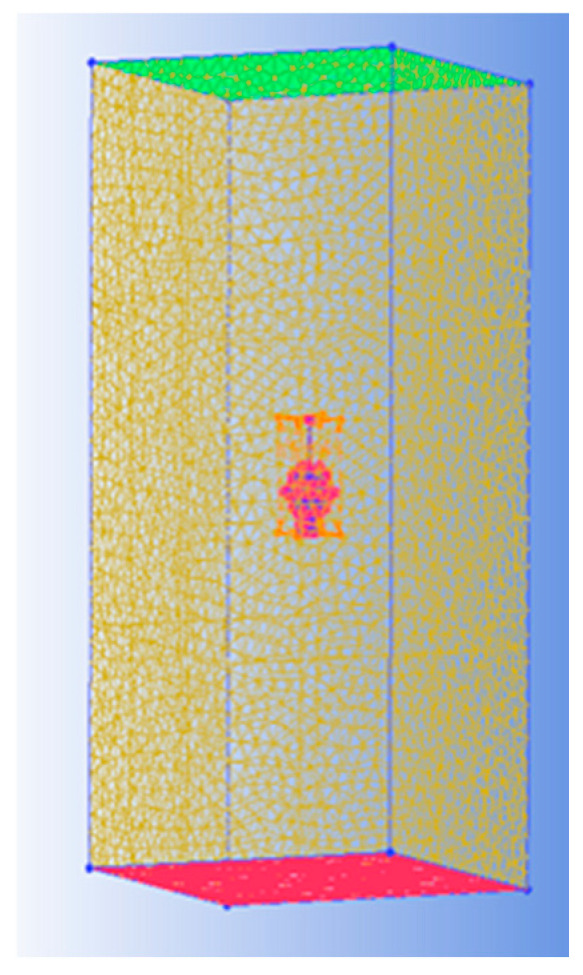
The computational domain and grid of DSIB (the number of grids is about 3.2 million).

To save time and cost, in this study, the hydrodynamic analysis is performed for the DSIB by using the CFD solver, FLUENT^®^ 6.3, instead of conducting hydrodynamic experiments. The Reynolds mean Navier–Stokes equations based on the SIMPLEC algorithm [15], along with the SST k-omega turbulence model [16], are adopted to study the hydrodynamic conditions of the DSIB body. The unstructured tetrahedral mesh for the geometry of the DSIB is also taken into consideration in this study. Gambit™ is used to discretize the flow domain into a finite set of control volumes [17,18]. As shown in Figure 3, the unstructured tetrahedron cells as the meshing grid are adopted to keep the meshes distributing reasonably and to make the meshes generate expediently.

**Figure 4:**
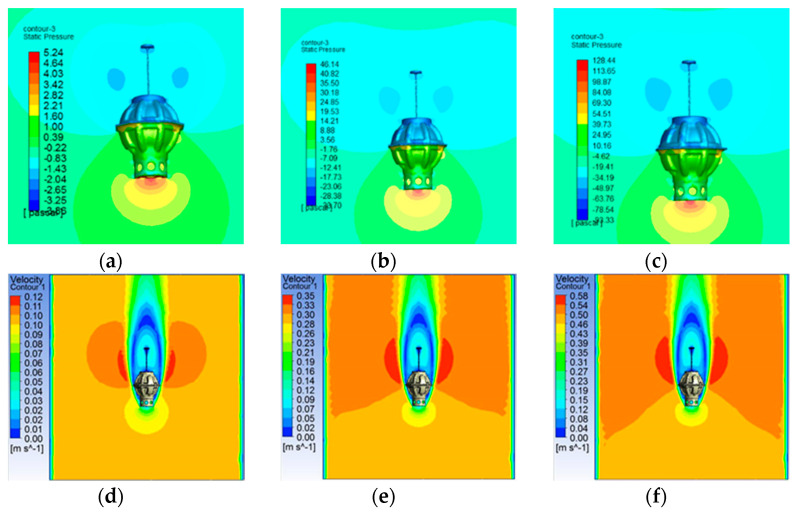
Static pressure contours and velocity vectors of the DSIB at different velocities in the diving process: diving surface pressure distribution of DSIB at different velocities: (**a**) 0.1 m/s; (**b**) 0.3 m/s; (**c**) 0.5 m/s; diving velocity field at different velocities: (**d**) 0.1 m/s; (**e**) 0.3 m/s; and (**f**) 0.5 m/s.

**Figure 5:**
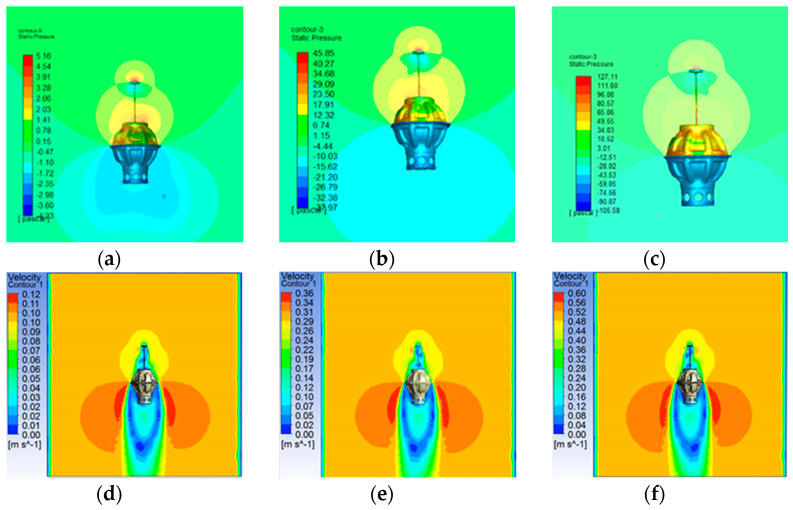
Static pressure contours and velocity vectors of the DSIB at different velocities in ascending process: ascending surface pressure distribution of DSIB at different velocities: (**a**) 0.1 m/s; (**b**) 0.3 m/s; (**c**) 0.5 m/s; ascending velocity field at different velocities: (**d**) 0.1 m/s; (**e**) 0.3 m/s; and (**f**) 0.5 m/s.

Before the analysis of the drag force conducting on DSIB, the flow field is analyzed. Figure 4 and Figure 5 show the simulation results of the diving and ascending motions; Figure 4a–c displays the pressure contours of the DSIB at diving velocities of 0.1, 0.3, and 0.5 m/s, respectively. The pressure on the DSIB increases with the diving velocity. The maximum pressure occurs at the base of the DSIB. The DSIB has a certain “vacuum region”, where the pressure is negative. As shown in Figure 4d–f, the water flows through the largest diameter of the protective shell and falls off. Hence, a distinct negative velocity region is created above the DSIB owing to the formation of large-scale vortices. The pressure contours of the DSIB in Figure 5a–c show that the maximum pressure is at the upper protective shell during the ascending motion. As the ascending velocity increases, the pressure on the DSIB also gradually increases. As the case of diving motion, and the ascending motion as well, it is noted in Figure 5d–f that the water flows through the largest diameter of the protective shell and falls off. However, a distinct negative velocity region is formed below the DSIB. The analysis results is given in Table 1.

### 2.3. Flow Field Analysis

Giving different flow velocities from 0.1 m/s to 0.5 m/s at the entrance of the CFD calculation domain, is used to simulate the floating or diving processes of the DSIB. The overall drag forces on the DSIB are obtained by CFD at these velocities in Table 2 and Table 3. The mean drag coefficients of the DSIB in ascending and diving processes are calculated to be *C_d-up_* = 0.74 and *C_d-down_* = 0.68.

## 3. DSIB Kinematics and Dynamics

The dynamic model of DSIB on the vertical plane is nonlinear and coupled, however, the linearization idea is used to linearize the DSIB dynamic model in this paper. Therefore, it is necessary to linearize the operating point of the model based on the following special assumptions:

(1) The total mass of the DSIB remains unchanged, and the center of mass and the center of buoyancy remain collinear in the axial direction—in the vertical plane, the DSIB system is regarded as a solid sphere, and only the movement in the vertical direction is considered; and
(2) Because the current below 200 m is very small under deep-sea environmental conditions, the current in the vertical plane can be ignored—the water resistance is linearized in this paper.

**Figure 6:**
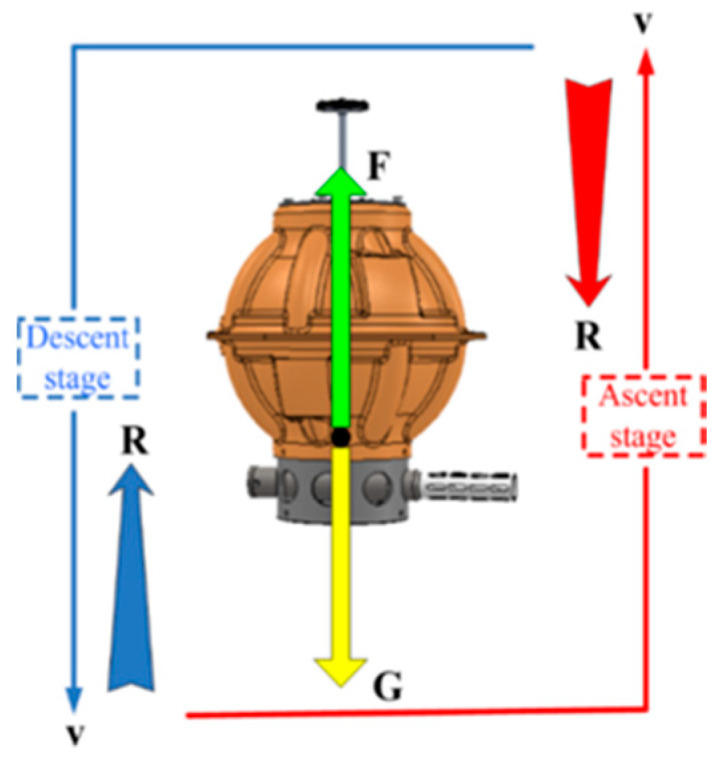
Significant forces acting on the DSIB during the ascent and descent processes in vertical plane.

According to the aforementioned special assumptions, the dynamic model of DSIB in the vertical plane is established. The DSIB floats or dives from the initial depth to the appointed depth at a speed of **v**. During the above operation process, the relevant force vector and velocity vector acting on the DSIB mainly include the buoyancy **F**, gravity **G**, resistance **R** and the motion speed of DSIB **v**, as shown in Figure 6. The ascent process is represented by the region marked by solid line, and the descent process is represented by the region of dotted line.

Assuming that the DSIB reaches an appointed depth of *h*, where *h* is a fixed value, the corresponding depth error can be calculated as $\hat{z}=z-h$, where *z* is the displacement of the DSIB in the ascent and descent processes. Different displacements of the DSIB in the vertical plane are produced by the hydraulic oil volume of the external bladder. When the DSIB hovers at an appointed depth, $\hat{z}=0$. According to the dynamic analysis of DSIB, the ascent and descent processes of the DSIB are expressed by the following force equilibrium equation for static condition in the vertical plane. Table 4 shows the description of the parameters in the DSIB kinetic equation.

(2) $$\left\{ \begin{array}{l} (M+M_{a})v^{\prime }=sign(v)[\rho_{\left(\hat{z}+h\right)}g(V_{f}-\Delta V+Q)-Mg]-\frac{1}{2}C_{d}A\rho_{\left(\hat{z}+h\right)}v\left|v\right|\\ \dot{\hat{z}}=v\\ M_{a}=\frac{2}{3}\pi \rho_{\left(\hat{z}+h\right)}r^{3} \end{array}\right.$$

where Δ*V* is the DSIB hull deformation. Due to the DSIB hull deformation caused by seawater pressure is much larger than that caused by seawater temperature, the DSIB hull deformation caused by seawater pressure is only considered in this paper [19]. By calculation, Δ*V* = (1.844 × 10^−^^11^)*P*_z_, *P_z_* is the pressure at the target depth *z* [19]. In terms of water-resistance calculation, the velocity component of the drag term is expressed as v|v| instead of *v^2^* [20]. The *sign* (*v*) is symbolic function. The downward motion of the DSIB is defined as a positive direction and the speed of the DSIB is positive. The *sign* (*v*) is expressed as follows:

(3) $$sign(v)=\left\{ \begin{array}{ll} -1, & v>0\\ 1, & v\leq 0 \end{array}\right.$$

In terms of Equation (2), the state–space representation of the DSIB system is expressed as follows:

(4) $$\left[\begin{array}{c} \dot{v}\\ \dot{\hat{z}} \end{array}\right]=\left[\begin{array}{cc} R_{1} & R_{2}\\ 1 & 0 \end{array}\right]\left[\begin{array}{c} v\\ \hat{z} \end{array}\right]+\left[\begin{array}{c} R_{3}\\ 0 \end{array}\right]Q+\left[\begin{array}{c} R_{2}\\ 0 \end{array}\right]h+\left[\begin{array}{c} R_{4}\\ 0 \end{array}\right]$$

where $R_{1}=-\frac{C_{d}A\rho_{\left(\hat{z}+h\right)}\left|v\right|}{2M}$, $R_{2}=-sign(v)\frac{\rho^{2}{}_{\left(\hat{z}+h\right)}gq}{M}$, $R_{3}=sign(v)\frac{\rho_{\left(\hat{z}+h\right)}g}{M}$, and $R_{4}=sign(v)(\frac{\rho_{\left(\hat{z}+h\right)}gV_{f}}{M}-g)$.

In the light of the state–space representation of the DSIB Equation (4), the movement state vector of the DSIB system is $x(t)={\left[\begin{array}{cc} v & \hat{z} \end{array}\right]}^{T}$, where *v* and $\hat{z}$ are state variables. The appointed *h* is considered as a constant value. Therefore, the state–space representation of the DSIB Equation (4) can be changed into the following formula:

(5) $$\left\{ \begin{array}{l} \dot{\hat{z}}=v\\ \dot{v}=R_{1}v+R_{2}\hat{z}+R_{3}Q+R_{2}h+R_{4} \end{array}\right.$$

In the given DSIB system model state parameter variables, the oil volume and depth of the outer oil capsule can be obtained by pull wire displacement sensor and pressure sensor, respectively. The submergence depth *z* and hydraulic oil volume of the external bladder *Q* can be obtained by pull wire displacement sensor and depth sensor, respectively [21].

Let $x_{1}=\hat{z}$, $x_{2}=v$, $y=x_{1}$, and u=Q, where *x*_1_ and *x*_2_ are depth error and motion velocity of the DISB, while *u* denotes control input. Thus, Equation (5) is expressed as follows:

(6) $$\left\{ \begin{array}{l} {\dot{x}}_{1}=x_{2}\\ {\dot{x}}_{2}=f(x_{1},x_{2})+bu+\Delta_{1} \end{array}\right.$$

where *b* is control gains, $b=R_{3}$; *f*(*x*_1_,*x*_2_) is the unknown nonlinear dynamics of the DISB,$f(x_{1},x_{2})=R_{1}x_{2}+R_{2}x_{1}$; ∆_1_ is unknown nonlinear disturbance of DISB, Δ_1_ = R_4_.

## 4. ITCS Depth-Controller Optimization Method Design

The inversion method is a recursive process. In this method, the high-order nonlinear system is divided into several low-order subsystems, and the appropriate Lyapunov function is selected to design and construct the appropriate virtual control law step by step. Finally, the real control law of the system is obtained to achieve the control purpose [22,23]. In the actual system control process, the system is often required to achieve the control requirements in a short time. In this regard, scholars have proposed the concept of time-constraint stability. The response speed and anti-interference ability of the system are improved by using the time-constraint stability strategy. Especially in recent years, with the development of time-constraint Lyapunov stability analysis and other theories, the application scope of time-constraint control method is broadened [24,25], and the control effect is further improved by combining with other algorithms, such as the inversion method and disturbance observer. In this paper, based on the inversion method, the inversion time-constraint stability (ITCS) controller is constructed by combining the inversion time-constraint control method with the technology of the nonlinear disturbance observer (NDO). The design process of the ITCS controller is given below.

**Lemma** **1** [26]**.** *Consider the following system*:

(7) $$\dot{x}=f(x),f(0)=0$$

where, $x\in R^{n}$, $f(\cdot ):U\to R^{n}$ is a continuous function from the domain *U* to the n-dimensional space *R*^n^. If there exist a continuous differentiable function V(x):U→R, *V* (x) is a positive definite function and *c* > 0 and λ∈(0,1), such that the following inequalities hold:

(8) $$\dot{V}(x)+c{\left(V(x)\right)}^{\lambda }\leq 0,x\in U\backslash \left\{0\right\}$$

Then, the Equation (8) is time-constraint stable.

(a) Design of nonlinear disturbance observer

According to the design theory of nonlinear disturbance observer, $\hat{i}$ is defined as the estimation of interference *i*, the estimation error is $s_{0}=i-\hat{i}$, the state equation of the DSIB system can be obtained as follows:

(9) $$\dot{x}(t)=f(x_{1},x_{2})+P_{1}(x)u+P_{2}(x)\Delta_{1}$$

where $P_{1}(x)={\left[\begin{array}{cc} 0 & b \end{array}\right]}^{T}$, $P_{2}(x)={\left[\begin{array}{cc} 0 & 1 \end{array}\right]}^{T}$.

The nonlinear disturbance observer is designed as follows:

(10) $$\left\{ \begin{array}{l} \hat{i}=n+c(x)\\ \dot{n}=J[{-f(x}_{1},x_{2})-P_{1}(x)u-P_{2}(x)(n+k(x))] \end{array}\right.$$

where $w(x)=w_{1}\hat{z}+w_{2}v$, *w*_1_ and *w*_2_ are constants, which are greater than 0.

Let $J=\frac{\partial c(x)}{\partial x}=[\begin{array}{cc} w_{1} & w_{2}] \end{array}$ is the observation gain. If the dynamic performance of the disturbance term changes slowly relative to the system, the dynamic equation of the disturbance observer satisfies ${\dot{s}}_{0}(t)+\frac{\partial c(x)}{\partial x}s_{0}(t)=0$. It can be obtained from Equation (10) $\dot{\hat{i}}=JP_{2}(x)s_{0}$, ${\dot{e}}_{0}=-w_{2}s_{0}$.

The Lyapunov positive definite function of the disturbance observer is defined as $V=\frac{1}{2}s_{0}{}^{2}$. The derivative is obtained as: $\dot{V}=s_{0}{\dot{s}}_{0}=-w_{2}s_{0}^{2}\leq 0$. It can be seen that the designed disturbance observer is stable. The controlled variable of the disturbance observer is: $W_{i}=\frac{1}{b}\hat{i}$.

(b) Design of inversion time-constraint stability controller

According to the state equation expression of the DSIB, based on the assumption that the DSIB reaches the predefined target depth *h*, where *h* is a fixed value, the associated depth error is set as $s_{1}=\hat{z}=z-h$, then

(11) $${\dot{s}}_{1}=\dot{\hat{z}}=\dot{z}-\dot{h}=v-\dot{h}$$

The first virtual control quantity is defined as: $\beta_{1}=-k_{1}s_{1}+\dot{h}$, *k*_1_ is a constant, which is greater than 0. Let $s_{2}=v-\beta_{1}$:

Step 1: The Lyapunov function *V*_1_ is defined as $V_{1}=\frac{1}{2}s_{1}{}^{2}$, the derivative is obtained as:

(12) $${\dot{V}}_{1}=s_{1}{\dot{s}}_{1}=s_{1}(v-\dot{h})=s_{1}(s_{2}+\beta_{1}-\dot{h})=-k_{1}s_{1}^{2}+s_{1}s_{2}$$

It can be seen from formula (12), when s_2_ = 0, ${\dot{V}}_{1}$ is negative definite. The subsystem satisfies the stability in the sense of Lyapunov, usually, $s_{2}\neq 0$ in an actual situation. Therefore, the next step of design is required.

Step 2: The Lyapunov function *V*_2_ is defined as follows: $V_{2}=V_{1}+\frac{1}{2}s_{2}{}^{2}$.The derivative of *S*_2_ is obtained as: ${\dot{s}}_{2}=\dot{v}-{\dot{\beta }}_{1}=f(x_{1},x_{2})+bu+\Delta_{1}-{\dot{\beta }}_{1}$.

The finite time-control method is used to design the state variable *s*_2_, and the expression of fuel controller *u* is obtained as follows:

(13) $$u=\frac{1}{b}(-f(x_{1},x_{2})-\Delta_{1}+{\dot{\beta }}_{1}-s_{1}-k_{2}sign(s_{2}){\left|s_{2}\right|}^{a})$$

where *k*_2_ is the normal number, the derivative is as follows:

(14) $${\dot{V}}_{2}=-k_{1}s_{1}^{2}+s_{1}s_{2}+s_{2}[f(x_{1},x_{2})+bu+\Delta_{1}-{\dot{\beta }}_{1}]$$

Substituting Equation (11) into Equation (14), we get:

(15) $${\dot{V}}_{2}=-k_{1}s_{1}^{2}+s_{1}s_{2}+s_{2}[-s_{1}-k_{2}sign(s_{2}){\left|s_{2}\right|}^{a}]=-k_{1}s_{1}^{2}-k_{2}sign(s_{2}){\left|s_{2}\right|}^{a+1}$$

The error derivative of the state quantity *v* can be calculated as:

(16) $${\dot{s}}_{2}=\dot{v}-{\dot{\beta }}_{1}=s_{0}(t)-k_{2}sign(s_{2}){\left|s_{2}\right|}^{a}-s_{1}=-k_{2}sign(s_{2}){\left|s_{2}\right|}^{a}+i_{1}(t)$$

If the balance point of the DSIB depth-control system is the origin, then the state of the DSIB system converges to the region ε_1_ within a finite time, where the bounded extremum of total interference *i_1_(t)* is *δ*, and δ≥0, $\varepsilon_{1}=\left\{s_{2}:\left|s_{2}\right|\leq {\left(\frac{k_{3}+\delta }{C}\right)}^{1/a}\right\},C>0,0<a<1$.

**Proof** **of** **Lemma** **1.** Choose Lyapunov function $V(s_{2})=\frac{1}{2}s_{2}{}^{2}$. □

(17) $$\begin{array}{l} \dot{V}(s_{2})=s_{2}{\dot{s}}_{2}=-k_{2}sign(s_{2}){\left|s_{2}\right|}^{a+1}+s_{2}i_{1}(t)\\ \leq -C{\left|s_{2}\right|}^{a+1}+\delta \left|s_{2}\right|\leq -k_{3}\left|s_{2}\right|\leq -k_{3}{\left(\frac{k_{3}+\delta }{C}\right)}^{1/a}\leq 0 \end{array}$$

The initial state of the DSIB depth-control system is divided into the following two situations:

(1) The initial state of the system is outside ε_1_, namely $s_{2}\in R-\varepsilon_{1}$, since $\dot{V}(s_{2})<0$. Then there exists *t_1_* > 0, such that $s_{2}(t_{1})\in \varepsilon_{1}^{b}$, where $\varepsilon_{1}^{b}$ represents the boundary of *ε*_1_;
(2) The initial state of the system is within ε_1_, namely $s_{2}\in \varepsilon_{1}$, when $t\geq t_{1}$, satisfying $s_{2}(t)\in \varepsilon_{1}$. Let $\theta =\underset{s_{2}\in \varepsilon_{1}^{b}}{\inf}\left|s_{2}\right|$ and $\sigma (s_{2})=C{\left|s_{2}\right|}^{a+1}+\delta \left|s_{2}\right|$. Then the following relationship can be obtained as:
  (18) $$\left\{ \begin{array}{l} \theta ={\left(\frac{k_{3}+\delta }{C}\right)}^{1/a}\\ \sigma (\theta )=k_{3}{\left(\frac{k_{3}+\delta }{C}\right)}^{1/a} \end{array}\right.$$

For $\forall s_{2}\in \varepsilon_{1}^{b}$, there exists $\dot{V}(s_{2})\leq -\sigma (\theta )<0$, given that *V*(*s*_2_) and *σ*(*s*_2_) are continuous functions, it can be seen from the properties of the continuous function, there exists *η* > 0 such that when $t\in [t_{1},t_{1}+\eta ]$, there exists $s_{2}(t)\in \varepsilon_{1}$. The initial state of the DSIB depth-control system has been proved.

Substituting Equations (16)–(18) into Equation (18), we get:

(19) $$\begin{array}{l} {\dot{V}}_{2}=-k_{1}s_{1}^{2}+s_{1}s_{2}+s_{2}{\dot{s}}_{2}\\ \leq -k_{1}s_{1}^{2}-s_{2}^{2}-k_{2}sign(s_{2}){\left|s_{2}\right|}^{a+1}+i_{1}s_{2}<0 \end{array}$$

The boundary-layer method is used to suppress the chattering caused by the depth controller. The function *sign*(·) in Equation (10) is replaced by the saturation function *sat* (·). The saturation function is expressed as follows:

(20) $$sat(\varphi /\lambda )=\left\{ \begin{array}{l} \varphi /\lambda ,\left|\varphi \right|\leq \lambda \\ sign(\varphi ),\left|\varphi \right|>\lambda \end{array}\right.$$

where λ > 0 is the thickness of the boundary layer.

According to the time-constraint stability controller design lemma and the proof of Lemma 1, the designed depth controller (12) is time-constraint stable.

The control effect of the ITCS depth controller mainly depends on the ITCS parameter setting. The ITCS parameter tuning is the core of the ITCS controller. But as the submergence depth increases, the complicated control processes caused by the pressure hull deformation and the change of seawater density appear. However, ITCS parameters are numerous and hard to adjust. The ITCS parameter tuning method fails to fully meet the requirements. An efficient ITCS parameter tuning method exerts a direct impact on the control effect and reliability of the depth positioning. In this paper, the parameter tuning method based on QGA and GACPSO algorithm are introduced to optimize the three key parameters (*k*_1_, *k*_2_, and *k*_3_) of the ITCS depth controller. When the deviation *e*(t) is produced by both the system input *r*(t) and system output *y*(t), the QGA or GACPSO algorithm is carried out, and the parameters of ITCS are regenerated. The DSIB system is controlled by the ITCS controller until *e*(t) makes the objective function of the control system producing a minimum value. The QGA or GACPSO algorithm stops running and the system achieves good controllability.

The task of parameter tuning is to find the optimal parameter combination of ITCS to minimize the fitness function. Considering the control error, dynamic characteristics and rapidity and stability of the system response, the objective function of the optimization is established by the Integral of Time-weighted Absolute value of the Error (ITAE) [27], which is expressed as follows:

(21) $$J=\int_{0}^{\infty }(w_{1}\left|e(t)\right|t+w_{2}\left|u(t)\right|)dt+w_{3}\left|\sigma \right|+w_{4}t_{r}$$

where *e*(t) is system error, *t* is simulation time, *u*(t) is nonlinear state error feedback controller output, σ is overshoot of the depth-control system, and *t_r_* is rise time. *w*_1_, *w*_2_, *w*_3_, and *w*_4_ are weighting coefficients.

### 4.1. QGA–ITCS Depth Controller

**Figure 7:**
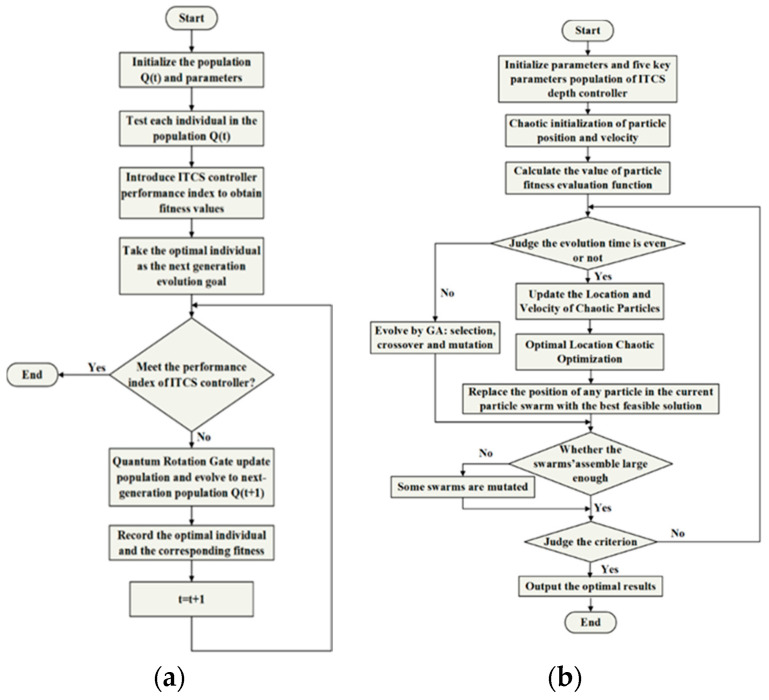
(**a**) Flow chart of parameter tuning process of the ITCS based on QGA algorithm; and (**b**) flow chart of parameter tuning process of the ITCS based on GACPSO algorithm.

Quantum genetic algorithm (QGA) is a combination of quantum computation theory and genetic algorithm principle. The concept of quantum computing was proposed by Beninff and Feynman [28]. In the late 1990s, Narayanan and Kuk-Hyun Han began to combine quantum theory with genetic algorithms, and successfully used them for multimodal function optimization and a class of combinatorial optimization problems and solution of the knapsack problem [29,30]. Thus, QGA not only improves the capability of global search, but also avoids the problem of premature convergence. The QGA can also effectively solve the problems of the Hamming cliff, calculate accuracy, and tackle other issues which appeared in the traditional genetic algorithm. In this paper, a fitness function of the DSIB system is defined, and the terms are weighted properly according to the demand of the actual DSIB system. Then a QGA algorithm, which represents chromosomes with quantum bits and realizes population evolution with the quantum rotation gate, is employed for multi-objective optimization of the ITCS depth controller. So, the parameter self-tuning of the ITCS depth controller can be achieved. The flow chart of ITCS control parameter optimization based on QGA algorithm is shown in Figure 7a. 

The implementation steps of the ITCS parameter tuning method based on QGA are as follows:

Step 1: The population *Q(t)* and parameters are initialized. $Q(t)=\{q_{1}^{t},q_{2}^{t},\cdots ,q_{n}^{t}\}$, where, $q_{j}^{t}$ is the *j*th Quantum Chromosome in the T generation population. The quantum bit of $q_{j}^{t}$ is expressed as follows:

(22) $$q_{j}^{t}=\left[\begin{array}{c} \alpha_{j1}^{t}\\ \beta_{j1}^{t} \end{array}\left|\begin{array}{c} \alpha_{j2}^{t}\\ \beta_{j2}^{t} \end{array}\right|\cdots |\begin{array}{c} \alpha_{jm}^{t}\\ \beta_{jm}^{t} \end{array}\right],j=1,2,\cdots ,n$$

where, *n* is the number of quantum chromosomes in the population, and m is the number of quantum bits that make up quantum chromosomes, which is the length of quantum chromosomes. In the optimization process of the ITCS controller, the three key parameters (*k*_1_, *k*_2_, and *k*_3_) need to be optimized. If each parameter is represented by 16-bit quantum bits, the length of the quantum bits chromosome is *m* = 80.

Step 2: The corresponding binary solution is obtained by observing the individual state in the population *Q*(*t*). $P(t)=\{x_{1}^{t},x_{2}^{t},\cdots ,x_{n}^{t}\}$, where $x_{j}^{t}=\{x_{j1}^{t},x_{j2}^{t},\cdots ,x_{jm}^{t}\}$, it is a binary string of length *m*. Each value of $x_{j}^{t}$ is 0 or 1, which is generated by a random number between 0 and 1 represented as r. If $\left|\alpha_{jk}^{t}\right|>r$, then $x_{jk}^{t}=1$, otherwise $x_{jk}^{t}=0$. The initialized key parameters (*k*_1_, *k*_2_, *k*_3_) are tested to obtain specific solutions.

Step 3: Each value of $x_{j}^{t}$ is converted to a real value within the range of the corresponding variable. According to the individual fitness evaluation function defined in Equation (21), the fitness of each chromosome is calculated.

Step 4: According to Equation (21), the adaptive value of each chromosome in the current population is calculated. The current optimal individual is found after comparison, and the binary corresponding to the optimal individual is stored in B(*t*). $B(t)=\{b_{1}^{t},b_{2}^{t},\cdots ,b_{n}^{t}\}$. The optimal individual and corresponding fitness are recorded. (The optimal individual is taken as the evolutionary target of the next generation).

Step 5: Whether the parameters of (*k*_1_, *k*_2_, and *k*_3_) are the optimal solution is determined. If the optimal solution is satisfied, the calculation exits; otherwise, the calculation continues. According to the value that does not satisfy the optimal solution, the (*k*_1_, *k*_2_, and *k*_3_) generated by the population are continuously acquired in real time, and the determined solutions are obtained.

Step 6: According to the Equation (23) and Table 5, the quantum rotation gate is used to update the population *Q*(*t*) of (*k*_1_, *k*_2_, and *k*_3_) chromosomes.

(23) $$\left[\begin{array}{c} \alpha_{i}{}^{\prime }\\ \beta_{i}{}^{\prime } \end{array}\right]=U(\Delta \theta_{i})\left[\begin{array}{c} \alpha_{i}\\ \beta_{i} \end{array}\right]$$

where ∆*θ_i_* (i = 1,2,...,m) is the rotation angle of each quantum bit toward |0〉 state or |1〉 state.${\left[\begin{array}{cc} \alpha_{i} & \beta_{i} \end{array}\right]}^{T}$ is the *i*th quantum bit of the quantum chromosome. Table 5 is the adjustment strategy of the quantum rotary gate. In Table 5, the $x_{jk}^{t}$ and $b_{jk}^{t}$ are the *k*th bit of the solution $x_{j}^{t}$ and current optimal solution $b_{j}^{t}$. $f(x_{j}^{t})$ and $f(b_{j}^{t})$ are fitness functions, respectively. ∆*θ_k_* is the rotation angle, that is the convergence speed of the control algorithm. $s(\alpha_{jk}^{t}\beta_{jk}^{t})$ is the direction function of the rotation angle.

Step 7: The number of iterations *t* is added to 1 and the algorithm is returned to step 2.

### 4.2. GACPSO–ITCS Depth Controller

Although the QGA algorithm avoids the defect shown in the traditional genetic algorithm of easily falling into the local optimum value, it often needs large-scale population to realize parameter tuning through many generations of cyclic computation, which spends more time and space on the optimization process. In this paper, the hybrid algorithm of complementary algorithm is created because of the disadvantages in QGA algorithm.

Particle swarm optimization (PSO) relates to swarm intelligence evolutionary computation methods with the global strategy and inspired nature. The basic idea derived from biological simulation on the foraging activities on bird populations, proposed jointly by the American psychologist Kennedy and electrical engineer Ebethart in 1995 [31], emerges according to swarm intelligence theory of computing technology. The algorithm mainly adopts the competition and cooperation mechanisms optimization guidance in the particles population searching process, and has good versatility and full retrieval ability. Particle swarm optimization (PSO) is simple a theory, quick in convergence, but likely to be “premature” at the initial stage. To locally improve the optimal solution of PSO, CPSO (chaotic particle swarm optimization) algorithm is introduced to enhance convergence accuracy and speed. The chaotic sequences of CPSO are generated by iteration, and the range of chaotic variables is corresponded to the value space of optimization variables by carrier mode [32]. Applicable to a variety of complex optimization problems and combinatorial optimization problems, the chaotic series are used to initialize the position and velocity of the particles. The diversity of the population and the ergodicity of the particle search are improved. 

In addition, genetic algorithm (GA) has strong full retrieval ability but the convergence accuracy is low. Considering both the advantages and disadvantages, genetic operators and the crossing-search methods are applied to PSO algorithm to avoid falling into locally optimal solutions. In this process, inertial weight and mutation methods are improved to balance the global and local search ability. At the same time, some swarms are mutated if the swarm population has evolved to a small enough space.

In this paper, the critical parameters of ITCS are operated by CPSO or GA of different parameters. Then, the optimal number is, respectively, selected by the genetic operators and chaotic series as a global optimum at every circulation, which improves the overall performance of the PSO algorithm. The flow chart of ITCS control parameter optimization based on GACPSO algorithm is shown in Figure 7b. The implementation steps of ITCS parameter tuning method based on GACPSO are as follows:

Step 1: The three key parameters (*k*_1_, *k*_2_, and *k*_3_) of the ITCS depth controller are encoded as five genes of an individual.

Step 2: The three key parameters (*k*_1_, *k*_2_, and *k*_3_) of population need to be initialized. Initiating a population is a random generation of a group of individuals. In order to increase the diversity of the population, N uniformly distributed random numbers r between [0, 1], and is the size of the population. The method for producing a single particle in any individual is expressed as follows:

(24) $$\varepsilon_{i}=\varepsilon_{i\_\min}+(\varepsilon_{i\_\max}-\varepsilon_{i\_\min})\times r\;i=1,2,3$$

The final initial population is [ε_1_, ε_2_, and ε_3_], that is, the three key parameters (*k*_1_, *k*_2_, and *k*_3_). The scale is N × 3.

Step 3: The particle position and velocity are randomly initialized according to the initial search interval. A three-dimensional vector corresponding to the three key parameters (*k*_1_, *k*_2_, and *k*_3_) is generated randomly. After mapping, a group of initial particles with random positions and velocities is obtained. The five parameters of each particle’s position variable are the three key parameters (*k*_1_, *k*_2_, and *k*_3_) of ITCS. According to Equation (21), the fitness value of the chaotic particle (the objective function value) is calculated.

Step 4: The number of particle iterations is increased and whether the number of evolutions is even or odd is determined.

(1) When the number of iterations is odd, the GA is introduced to select, cross and mutate the particles to update the velocity and position of the particles.
  (a) After selecting the appropriate initial population, it is necessary to select the optimal individual among these values. The individual fitness value of the parameter is calculated. Based on the fitness value, the good individual is selected to inherit the next generation according to certain rules;
  (b) The crossover probability *P*_c_ and the mutation probability *P*_m_ of the genetic algorithm can be automatically changed with individual fitness value. The selected cross mutation probability is adaptively adjusted as follows:
    (25) $$P_{c}=\{\begin{array}{c} p_{c1}-\frac{\left(f-f_{avg})(p_{c1}-p_{c2}\right)}{f_{\max-}f_{avg}},{}^{}f\geq f_{avg}\\ p_{c1},f<f_{avg} \end{array}$$
    (26) $$P_{m}=\{\begin{array}{c} p_{m1}-\frac{\left(f^{*}-f_{avg})(p_{m1}-p_{m2}\right)}{f_{\max-}f_{avg}},{}^{}f^{*}\geq f_{avg}\\ p_{m1},f^{*}<f_{avg} \end{array}$$
    where *f_max_* is the maximum fitness value in the population. *f_avg_* is the mean fitness value; *f* and f* are the fitness value of the larger individual and the fitness value of the mutant individual in the selected two crossover individuals, respectively. *p*_c1_ and *p*_c2_ were the maximum and minimum values of cross probability cues, and *p*_m1_ and *p*_m2_ were the maximum and minimum values of mutation probability cues.
(2) When the number of iterations is even, the CPSO is introduced to update the velocity and position of the particles.
  (a) Let $z_{i}={\left(z_{i1},z_{i2},\cdots ,z_{id}\right)}^{T}$ is the d-dimensional vector of the *i*th particle. $v_{i}={\left(v_{i1},v_{i2},\cdots ,v_{id}\right)}^{T}$ is the flight speed of the *i*th particle. $p_{io}={\left(p_{io1},p_{io2},\cdots ,p_{iod}\right)}^{T}$ is the optimal position of the *i*th particle. $p_{go}={\left(p_{go1},p_{go2},\cdots ,p_{god}\right)}^{T}$ is the optimal location for the entire particle swarm search. Particle optimal position *pi_o_* and particle global optimal position *pg_o_* are updated: If the particle fitness is better than the individual extreme value *pi_o_*, the new position is set to the particle optimal position *pi_o_*; if the particle fitness is better than the global extremum *pg_o_*, then the new position is set to the particle global optimal position *pg_o_*. According to Equations (27) and (28), the velocity and position of particles are updated, respectively.
    (27) $$v_{id}^{k+1}=wv_{id}^{k}+c_{1}r(pi_{o}^{k}-z_{id}^{k})+c_{2}r(pg_{o}^{k}-z_{id}^{k})$$
    (28) $$z_{id}^{k+1}=z_{id}^{k}+z_{id}^{k+1}$$
    where *c*_1_ and *c*_2_ are acceleration factor; r_1_ and r_2_ are random numbers between [0, 1]; *w* is the inertia factor.
  (b) Chaos optimization is carried out for the optimal position. The optimal position is mapped to the domain of the Logistic equation [0, 1] by using the equation $y_{i}=(pg_{oi}-a_{i})/(b_{i}-a_{i})$(*i* = 1,2,...,d). The chaotic variable sequences $y_{i}^{\left(n\right)}(n=1,2,\cdots )$ are iteratively generated by using the Logistic equation, and is returned to the original solution space by inverse mapping $pg_{oi}{}^{\left(n\right)}=a_{i}+(b_{i}-a_{i})y_{i}^{\left(n\right)}$. The following formula can be obtained $p_{go}{}^{\left(n\right)}=(p_{go1}{}^{\left(n\right)},p_{go2}{}^{\left(n\right)},\cdots ,p_{god}{}^{\left(n\right)}),{}^{}(n=1,2,\cdots )$, where [*a_i_*, *b_i_*] is the feasible domain of the *i*-dimensional independent variable. In the original solution space, the fitness of each feasible solution $p_{goi}{{}^{\left(n\right)}}^{}(n=1,2,\cdots )$ experienced by the chaotic variable is calculated, and the best feasible solution is obtained, thus the position of any particle in the current particle swarm is replaced.

Step 5: According to the swarm assemble of particles in the process of searching the optimal solution, if the aggregation degree of particles exceeds a certain threshold, a certain number of particles is mutated according to equation $z_{i}{=}^{}p_{io}\times^{}(1^{}{+}^{}0.5\mu )$,in which μ is a random vector obeying (0, 1) normal distribution.

Step 6: The fitness value is determined again. Particle optimal position *pi_o_* and particle global optimal position *pg_o_* are determined again.

Step 7: Identifying whether the number of iterations meets the requirements is determined; if so, turn to Step 7; otherwise, turn to Step 4.

Step 8: The optimal value of the three key parameters (*k*_1_, *k*_2_, and *k*_3_) is obtained, respectively.

## 5. Results and Analysis

**Figure 8:**
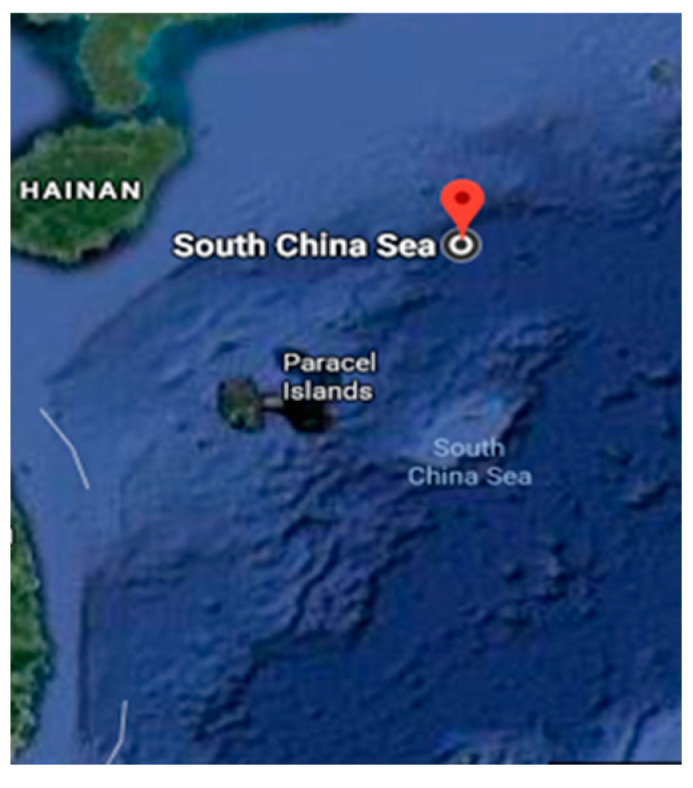
Position of the DSIB deployed in the South China Sea (18.35° N, 114.35° E).

Due to the high cost and the long experiment period of sea trials, a hardware-in-loop experimental system is used for experiments in this study. The system can simulate the change of seawater pressure with depth in real time when the DSIB is in operation and verify the effectiveness and stability of the depth-keeping control method. Thus, it provides a reliable reference for sea trials. Because the DSIB was deployed in two positions of the South China Sea (18.35° N, 114.35° E) in July 2018 (Figure 8). During the hardware-in-the-loop system experiments, the deployed sea area values of the depth, conductivity and temperature were further used to calculate the seawater density. The depth rate is used to evaluate the operating speed of the DSIB at the initial moment. In this paper, the average operating speed of DSIB system at the initial moment is about 0.5 m/s during the floating and submerging processes. In order to fully evaluate the control performance of the proposed QGA–ITCS and GACPSO–ITCS depth controller for the DSIB in the South China Sea, the simulation analysis and hardware-in-the-loop system experimental platform test are divided into shallow water area and deep water area with the depth of 2000 m as the demarcation point. 

The non-dynamic parameters of the DSIB for simulation were shown in Table 6. In order to compare the performance of QGA and GACPSO in searching the optimal parameters of ITCS depth controller, the same initial parameters were set as follows: Population size = 40; Generation times = 100.

The same initial observation gain parameters of ITCS were set as follows: *g*_1_ = 2, and *g*_2_ = 18.

**Figure 9:**
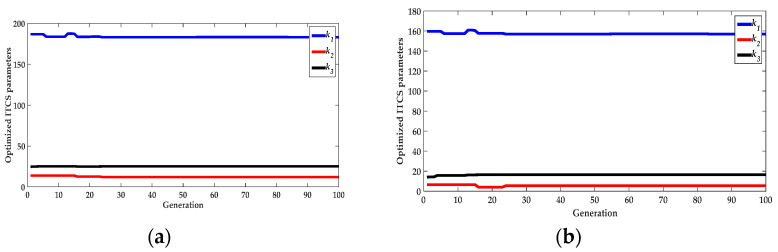
(**a**) Convergent process of the three key parameters (*k*_1_, *k*_2_, and *k*_3_) of the optimized ITCS by QGA method in the ascent process; and (**b**) convergent process of the three key parameters (*k*_1_, *k*_2_, and *k*_3_) of the optimized ITCS by QGA method in the descent process.

For the QGA–ITCS depth controller, the convergent effect of the ITCS parameters were optimized by the QGA method in Figure 9. The desirable parameters were set as follows:

(1) QGA parameters: The binary length of each variable = 20;
(2) Optimized ITCS parameters:

In ascent process: *k*_1_ = 183.212, *k*_2_ = 12.079, and *k*_3_ = 25.167.

In descent process: *k*_1_ = 156.856, *k*_2_ = 5.397, and *k*_3_ = 16.351.

**Figure 10:**
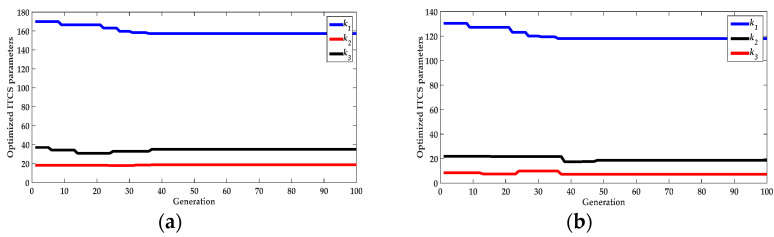
(**a**) Convergent process of the three key parameters (*k*_1_, *k*_2_, and *k*_3_) of the optimized ITCS by GACPSO method in ascent process; and (**b**) convergent process of the three key parameters (*k*_1_, *k*_2_, and *k*_3_) of the optimized ITCS by GACPSO method in descent process.

For the GACPSO–ITCS depth controller, the convergent effect of the optimized ITCS parameters by the GACPSO method was shown in Figure 10. The desirable parameters were set as follows:

(1) GACPSO parameters: Acceleration factor *c*_1_ = 2, *c*_2_ = 2; Inertia factor *w* = 0.6.
(2) Optimized ITCS parameters:

In ascent process: *k*_1_ = 157.322, *k*_2_ = 18.765, and *k*_3_ = 35.252.

In descent process: *k*_1_ = 118.037, *k*_2_ = 7.247, and *k*_3_ = 18.521.

Relevant simulations have been conducted to validate the proposed depth-control method compared to that of a standard ITCS controller. For the ITCS depth controller, the desirable parameters were as follows: 

In ascent process: *k*_1_ = 200, *k*_2_ = 6, and *k*_3_ = 10.

In descent process: *k*_1_ = 180, *k*_2_ = 8, and *k*_3_ = 20.

**Figure 11:**
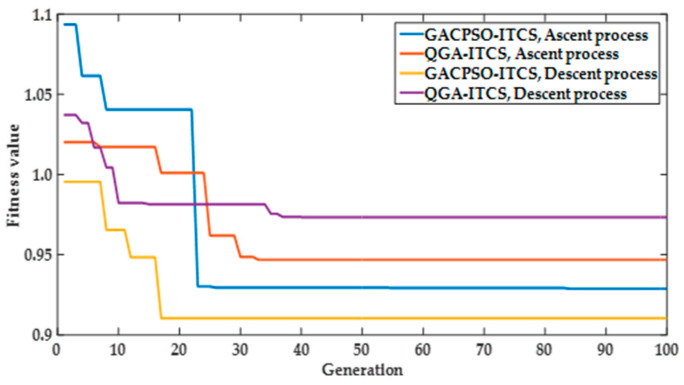
The performance index of fitness function change curve of the optimized ITCS by QGA and GACPSO method in ascent and descent processes.

As can been seen from Figure 11, simulations are performed using the QGA and GACPSO to compare the convergence characteristics. With the same environment and conditions, it can be easily found that the convergence tendency of GACPSO is faster than the QGA. Meanwhile, GACPSO can also obtain more accuracy or better fitness value than QGA in searching the optimal parameters of ITCS depth controller. In other words, GAGAC–PSO has better convergence characteristic in searching the optimal parameters of the ITCS depth controller.

### 5.1. Simulation Results and Analysis

In order to further verify the control effect of the designed depth controller, the control result is compared with that of the traditional PID depth controller by the simulation. The mean depth steady-state error and settling time are used as the control performance evaluation index of depth controller to evaluate the depth-control accuracy of DSIB system in this paper. The main simulation results of the ascent and descent processes are shown in Table 6. In the ascent process, the optimized ITCS depth controller enables the DSIB to reach an appointed depth of 500 m within 230 s, and the mean depth steady-state error is less than 2.7 m. In the diving process, the optimized ITCS depth controller takes about 240 s to reach an appointed depth of 3200 m, and the mean depth steady-state error is less than 2.5 m.

Scenario 1: Restriction to above 2000 m.

**Figure 12:**
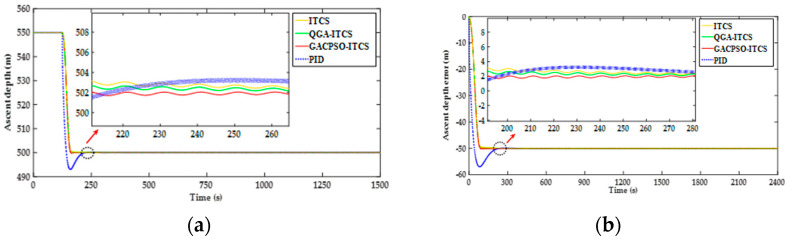
(**a**) Depth simulation response curve comparison chart of the ITCS controller after parameter optimization tuning by QGA and GACPSO method in ascent process; and (**b**) depth error simulation response curve comparison chart of the ITCS controller after parameter optimization tuning by QGA and GACPSO method in ascent process.

The simulation scenario of the shallow water area is as follows: assumed that the DSIB floats at an initial velocity of 0.5 m/s from an underwater depth of 550 m, and hovers at an appointed depth of 500 m. The simulation results of the depth and the depth error for the controllers, including PID depth controller, ITCS depth controller, QGA–ITCS, and GACPSO–ITCS depth controller, are illustrated in Figure 12.

As shown in Figure 12, the DSIB is affected by the joint interference of the water resistance and the net buoyancy change during the floating process. Under such circumstances, the depth-keeping control process of the DSIB at an appointed depth of 550 m is achieved by the PID depth controller, the ITCS depth controller, QGA–ITCS and GAC–PSO–ITCS depth controller. The settling time and mean depth steady-state error are used to describe the control performance index of the depth-keeping control process in this paper. However, the aforementioned depth-keeping controllers are different in terms of the settling time and depth steady-state error. In the ascending process, the settling time and mean depth steady-state error with ITCS controller are 225 s and 2.7 m, respectively, which are less than those with PID controller. Under the action of ITCS depth controller based on QGA and GACPSO strategy, the settling time of the aforementioned two depth-keeping controllers is shortened by 11 s and 19 s compared with that of ITCS depth controller. When the DSIB system reaches the target depth, the mean depth steady-state error of ITCS depth controller based on QGA and GACPSO strategy is reduced by 11% and 30%, compared with that of ITCS depth controller.

Scenario 2: Restriction to depth between 2000 m and 4000 m.

**Figure 13:**
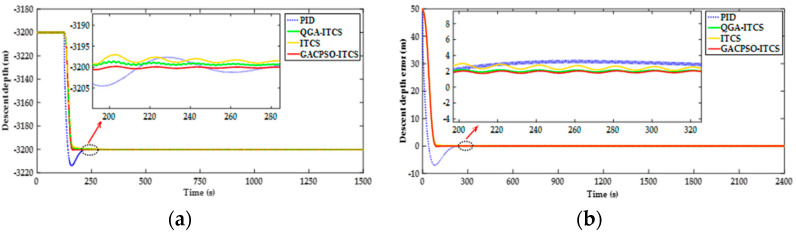
(**a**) Depth simulation response curve comparison chart of ITCS controller after parameter optimization tuning by QGA and GACPSO method in descent process; and (**b**) depth error simulation response curve comparison chart of ITCS controller after parameter optimization tuning by QGA and GACPSO method in descent process.

The simulation scenario of the deep water area is as follows: assumed that the DSIB descends at an initial velocity of 0.5 m/s from an underwater depth of 3150 m, and hovers at an appointed depth of 3200 m. Figure 13 shows the simulation results of the depth and the depth error for the PID depth controller, ITCS depth controller, QGA–ITCS and GAC–CPSO–ITCS depth controller. The results are similar to the diving process. In Figure 13, we can see that, under the same influence of external interference in the diving process, by employing the ITCS depth controller compared to the PID depth controller, the settling time is reduced from 262 s to 236 s and the mean depth steady-state error is reduced from 3.3 m to 2.5 m. The mean depth steady-state error of the ITCS depth controller based on QGA and GACPSO strategy is 16% and 28% less than that of the ITCS depth controller, respectively.

In summary, compared with PID depth controller, the ITCS depth controller and optimized ITCS depth controller have higher control accuracy and robustness under the same external disturbance of the ascending and diving process. Besides, the GACPSO–ITCS depth controller takes about less time to reach the desired depth, which is faster than the ITCS depth controller and QGA–ITCS depth controller in the ascending and diving process. The depth steady-state errors are less for the GACPSO–ITCS depth controller than that for the ITCS depth controller and QGA–ITCS depth controller, which means that the GACPSO–ITCS depth controller is significantly better than the other two depth controllers.

### 5.2. Results of the Hardware-in-the-Loop System Experiments

**Figure 14:**
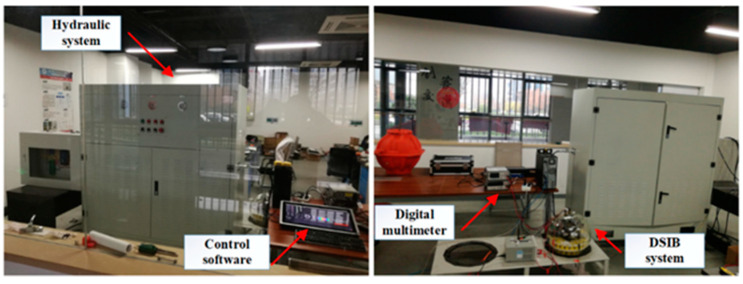
Hardware-in-the-loop system experimental platform: mainly includes hydraulic device, simulation control software, digital multimeter, and DSIB system.

In order to verify the effectiveness and reliability of the designed hover control strategy, this paper uses the established 0–60 MPa high-pressure environment hardware-in-the-loop system experimental platform to complete the experimental verification process of the he proposed depth-keeping optimization controller, as shown in Figure 14. The hardware-based experiment platform mainly includes hydraulic device, simulation control software, digital multimeter, and DSIB system.

**Figure 15:**
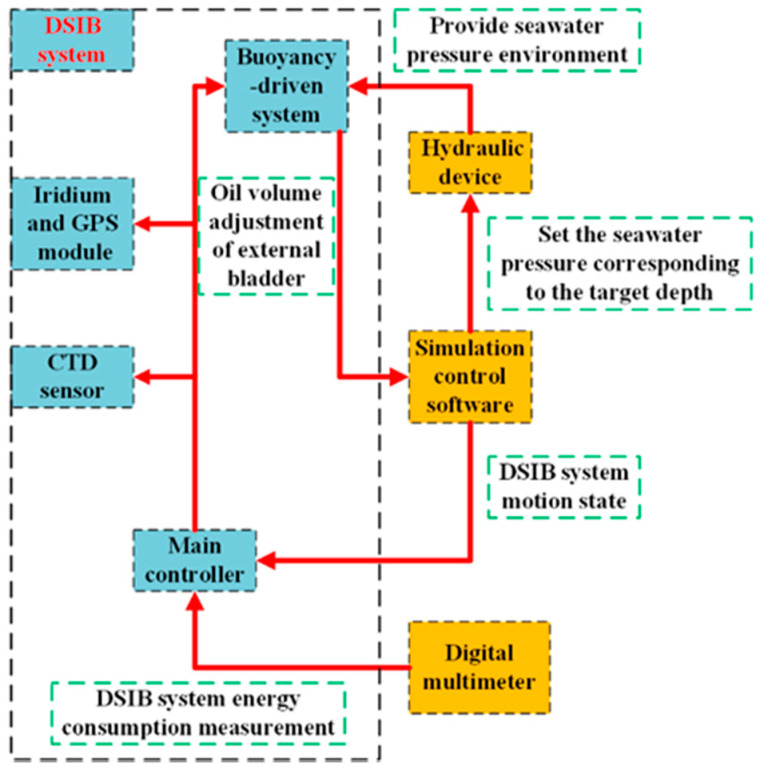
Schematic diagram of the structural relationship of the hardware-in-the-loop system experiment platform: mainly includes hydraulic device, simulation control software, digital multimeter, and DSIB system.

The schematic diagram of the structure of the hardware-in-the-loop system experiment platform is shown in Figure 15. The action of the buoyancy-driven system is controlled by the main controller of the DSIB system, and the oil volume parameters of the external bladder is sent to the control software of the computer. The acceleration, velocity and depth of DSIB system at the next time are calculated by the control software according to the current depth, seawater temperature, and density data. Then the calculated motion state of the DSIB system is used as the operation reference. At the same time, the hydraulic device is controlled by the computer to generate the pressure value required by the current depth of DSIB system, so that the working process of buoyancy-driven system is consistent with the real seawater pressure. Relevant information such as specified target depth and depth-control algorithm and control parameters are input in the computer. According to the aforementioned input information, the hydraulic device can simulate the current depth pressure and track the specified target depth pressure. Finally, the specified target depth pressure tracking curve is processed to obtain the depth-control process curve, and the digital multimeter is used to measure the voltage and current of the DSIB system power supply in the aforementioned control process in real time, thereby the energy consumption of the DSIB system is obtained.

**Figure 16:**
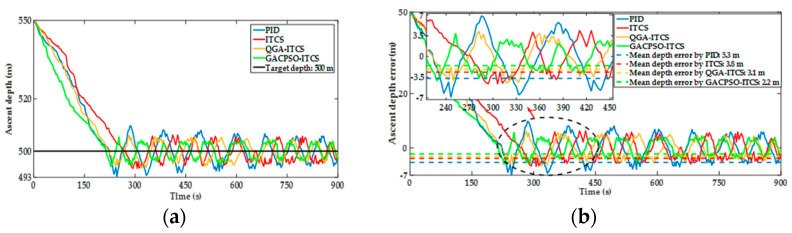
(**a**) Depth experimental response curve comparison chart of ITCS controller after parameter optimization tuning by QGA and GACPSO method in ascent process; and (**b**) depth error experimental response curve comparison chart of ITCS controller after parameter optimization tuning by QGA and GACPSO method in ascent process.

**Figure 17:**
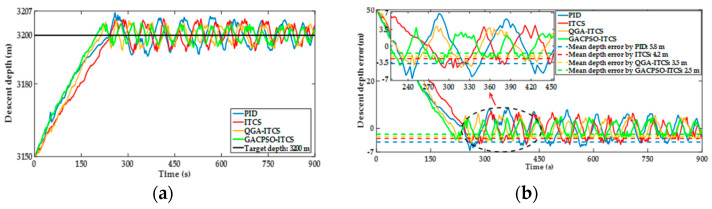
(**a**) Depth experimental response curve comparison chart of ITCS controller after parameter optimization tuning by QGA and GACPSO method in descent process; and (**b**) depth error experimental response curve comparison chart of ITCS controller after parameter optimization tuning by QGA and GACPSO method in descent process.

The experimental results of the ascent and descent processes from the hardware-in-the-loop system are shown in Figure 16 and Figure 17. The same depth error change trend is illustrated in the experimental and simulation results. The comparison of the main experimental results in the ascent and descent processes are shown in Table 7. Compared with the simulation results, the DSIB took more settling time to reach the appointed depth, and had more mean depth steady-state error in the hardware-in-the-loop system experiment. In the ascent process or descent process, the mean depth steady-state error tended to a small neighborhood near 0 within 450 s, and the mean depth steady-state error was less than 6 m. 

In Table 7, during the ascent process, the PID depth controller makes the DSIB system reach the appointed depth of 500 m after adjusting 413 s. The mean depth steady-state error is 5.3 m, and there is still a small oscillation after reaching the appointed depth. Whereas the ITCS depth controller takes about 347 s to reach an appointed depth of 500 m, and the mean depth steady-state error is 3.8 m. Furthermore, the ITCS depth controller based on QGA and GACPSO method reaches the appointed depth of 500 m after adjusting 313 s and 292 s. Compared with the ITCS depth controller, the mean depth steady-state error is reduced by 18.4% and 42.1%. The control effect of designed depth controller during the descent process of the DSIB system is similar to that of the ascent process. Compared with the control results of ITCS depth controller, the depth steady-state error of the ITCS depth controller based on QGA and GACPSO method is reduced by 16.7% and 40.5%. In summary, under the same external interference conditions in the ascent and descent processes, the control effect of the designed QGA–ITCS and GACPSO–ITCS depth controller are obviously better than that of PID depth controller in terms of settling time and mean depth steady-state error. The feasibility and effectiveness of the designed ITCS depth controller based on QGA and GACPSO method are verified. The parameters obtained by using GACPSO method to optimize the ITCS depth controller have the smallest mean depth steady-state error during the ascending and diving process, and its control accuracy is higher.

## 6. Conclusions

In this paper, in order to establish a more accurate dynamic model for DSIB, the hydrodynamic properties of the DSIB were estimated by using the turbulence models of SST k-omega. Thus, the analysis of the DSIB’s drag coefficients, pressure contours, and velocity vectors were obtained. Based on the analyzed hydrodynamic coefficients of the CFD simulation, the dynamic model of the DSIB was established. To reduce effects of the aforementioned external disturbance factors on hovering motion behaviors of the DSIB, the NDO was used to suppress and compensate the error caused by the external disturbance. The ITCS was used to drive the state of the depth-keeping control system to force the depth error to an arbitrarily small neighborhood of zero within a specified time limit. In order to obtain the optimal ITCS depth-controller parameters combination and improve the quality of dynamic adjustment, the ITCS parameters were optimized by using QGA strategy. To solve the problems of high space consumption and further improve the low speed and accuracy of global search in the parameter optimization process of the single QGA algorithm, an effective optimization hybrid strategy based on CPSO and GA was proposed to optimize ITCS depth-control parameters by the global optimums only. The simulations and hardware-in-the-loop system experimental results indicated that the GACPSO–ITCS has better depth positioning precision, depth-control system response speed and convergence stability compared with ITCS and QGA–ITCS depth controller. The designed depth-keeping control strategy provides some reference for ITCS parameters optimization of depth-control process. The proposed depth controller can ensure a high-precision data acquisition on the ocean environment observation of a target depth. Due to the limitation of time and test conditions, the hydrodynamic coefficients of the DSIB system are mainly obtained by CFD simulation. In order to further verify the feasibility of the hydrodynamic coefficients calculated from CFD, future research work can compare the obtained hydrodynamic coefficients through the analytical relations and tank drag experiments, respectively. Although the hardware-in-the-loop system experiments analysis shows that the depth positioning can be achieved by the designed depth controller with high accuracy and fast response, further work is necessary to test the full applicability of the designed depth controller and conduct the at-sea experiments in realistic engineering conditions.

## Figures and Tables

**Table 1:** CFD analysis results of DSIB in the diving process and the ascending process.

Velocity (m/s)	0.1		0.3		0.5	
Maximum Pressure (Pa)	Positive Pressure	Negative Pressure	Positive Pressure	Negative Pressure	Positive Pressure	Negative Pressure
Ascending motion	5.16	4.23	45.85	37.97	127.11	105.58
Diving motion	5.24	3.86	46.14	33.7	128.44	93.33

**Table 2:** Drag force acts on the DSIB in ascending process.

Motion Velocity of DSIB (m/s)	0.1	0.2	0.3	0.4	0.5
Drag Force (N)	1.16	4.63	10.37	18.51	28.83

**Table 3:** Drag force acts on the DSIB in diving process.

Motion Velocity of DSIB (m/s)	0.1	0.2	0.3	0.4	0.5
Drag Force (N)	1.01	3.96	8.83	15.62	24.34

**Table 4:** Description of the parameters in the DSIB kinetic equation.

Parameter	Units	Description	Parameter	Value	Units	Description
$\rho_{\hat{z}+h}$	kg/m^3^	Seawater density at a set depth scope	*M*	52.39	kg	Total mass of the DSIB
$\hat{z}$	m	Depth error	*g*	9.8	m/s^2^	Acceleration due to gravity
*h*	m	Appointed depth	*V_f_*	0.0504	m^3^	Volume of the DSIB
*v*	m/s	Motion velocity of the DSIB	*C_d-up_*	0.73		Drag coefficient as the DSIB floating
*M_a_*	kg	Added mass of the DSIB at a set depth scope	*C_d-down_*	0.66		Drag coefficient as the DSIB submerging
Δ*V*	m^3^	DSIB hull deformation	*A*	0.301	m^2^	Projected area of DSIB hull
*Q*	m^3^	Hydraulic oil volume of external bladder	*r*	0.216	m	Radius of the spherical pressure hull

**Table 5:** Non-dynamic constants of the DSIB.

$x_{jk}^{t}$	$b_{jk}^{t}$	$f(x_{j}^{t})\geq f(b_{j}^{t})$	$\Delta \theta_{k}$	$s(\alpha_{jk}^{t}\beta_{jk}^{t})$			
				$\alpha_{jk}^{t}\beta_{jk}^{t}>0$	$\alpha_{jk}^{t}\beta_{jk}^{t}<0$	$\alpha_{jk}^{t}=0$	$\beta_{jk}^{t}=0$
0	0	False	0	0	0	0	0
0	0	True	0	0	0	0	0
0	1	False	0	0	0	0	0
0	1	True	0.01π	−1	+1	±1	0
1	0	False	0.01π	−1	+1	±1	0
1	0	True	0.01π	+1	−1	0	±1
1	1	False	0.01π	+1	−1	0	±1
1	1	True	0.01π	+1	−1	0	±1

**Table 6:** Comparison of main simulation results in the ascent and descent processes.

Direction of Motion	Depth- Control Strategy	Controller Parameters Setup	Mean Depth Steady-State Error (m)	Settling Time (s)
Ascent process	PID	*k*_p_ = 260, *k*_i_ = 80, *k*_d_ = 50	3.5	243
	ITCS	*k*_1_ = 200, *k*_2_ = 6, *k*_3_ = 10	2.7	225
	QGA–ITCS	*k*_1_ = 183.212, *k*_2_ = 12.079, *k*_3_ = 25.167	2.4	214
	GACPSO–ITCS	*k*_1_ = 156.856, *k*_2_ = 5.397, *k*_3_ = 16.351	1.9	206
Descent process	PID	*k*_p_ = 8, *k*_i_ = 1.5, *k*_d_ = 30	3.3	262
	ITCS	*k*_1_ = 180, *k*_2_ = 8, *k*_3_ = 20	2.5	236
	QGA–ITCS	*k*_1_ = 156.856, *k*_2_ = 5.397, *k*_3_ = 16.351	2.1	227
	GACPSO–ITCS	*k*_1_ = 118.037, *k*_2_ = 7.247, *k*_3_ = 18.521	1.8	218

**Table 7:** Comparison of main hardware-in-the-loop system experiment results in ascent and descent process.

Direction of Motion	Depth- Control Strategy	Controller Parameters Setup	Mean Depth Steady-State Error (m)	Settling Time (s)
Ascent process	PID	*k*_p_ = 260, *k*_i_ = 80, *k*_d_ = 50	5.3	419
	ITCS	*k*_1_ = 200, *k*_2_ = 6, *k*_3_ = 10	3.8	347
	QGA–ITCS	*k*_1_ = 183.212, *k*_2_ = 12.079, *k*_3_ = 25.167	3.1	313
	GACPSO–ITCS	*k*_1_ = 156.856, *k*_2_ = 5.397, *k*_3_ = 16.351	2.2	292
Descent process	PID	*k*_p_ = 8, *k*_i_ = 1.5, *k*_d_ = 30	5.8	434
	ITCS	*k*_1_ = 180, *k*_2_ = 8, *k*_3_ = 20	4.2	356
	QGA–ITCS	*k*_1_ = 156.856, *k*_2_ = 5.397, *k*_3_ = 16.351	3.5	331
	GACPSO–ITCS	*k*_1_ = 118.037, *k*_2_ = 7.247, *k*_3_ = 18.521	2.5	315

## Data Availability

Not applicable.
